# Supplementary material for: MARBLE: interpretable representations of neural population dynamics using geometric deep learning
Source: Nat Methods. 2025 Feb 17;22(3):612–20. doi: 10.1038/s41592-024-02582-2 (PMC11903309; doi:10.1038/s41592-024-02582-2)
Supplement: Supplementary file 1 — Supplementary Notes 1–3, Figs. 1–5 and Tables 1–3. [file 41592_2024_2582_MOESM1_ESM.pdf]

nature methods

Supplementary information https://doi.org/10.1038/s41592-024-02582-2

MARBLE: interpretable representations of neural population dynamics using geometric deep learning

In the format provided by the authors and unedited

Contents

1 Supplementary Notes **2**
1.1 Supplementary Note 1: Pseudo code of MARBLE algorithm 2
1.2 Supplementary Note 2: Orientation ambiguity of local coordinate frames 2
1.3 Supplementary Note 3: Mathematical construction of the gradient filters 2

2 Supplementary Figures **4**

3 Supplementary Tables **8**

1 Supplementary Notes

1.1 Supplementary Note 1: Pseudo code of MARBLE algorithm

We implemented MARBLE architecture with Pytorch Geometric[1]. The general algorithm is as follows.

Algorithm 1 MARBLE

Input: d-dimensional vector field samples $\mathbf{F} = (\mathbf{f}_1, \ldots, \mathbf{f}_n)$
connection Laplacian $\mathcal{L}$
derivative filters $\mathcal{D}_i^{(q)}$ for $i \in \{1, \ldots, n\}$ and $q \in \{1, \ldots, m\}$
derivative order p

Output: Latent vectors $\mathbf{z}_i$ for all $i \in \{1, \ldots, n\}$

$\mathbf{F} \leftarrow e^{\tau\mathcal{L}}\mathbf{F}$ ▷ Apply diffusion layer (optional)
$\mathbf{h}^{(0)} \leftarrow \mathbf{f}_i$
for $1 \leq l \leq p$ **do** ▷ Loop over filter orders
  $\nabla h_q^{(l)} = \left(\mathcal{D}^{(1)}(h_q^{(l)}), \ldots, \mathcal{D}^{(m)}(h_q^{(l)})\right)^T$ ▷ Compute filters
  $\mathbf{h}^{(l)} \leftarrow \text{concat}\left(\mathbf{h}^{(l-1)}, \nabla h_1^{(l)}, \ldots, \nabla h_m^{(l)}\right)$ ▷ Concatenate derivatives
end for
$\mathbf{h}^{(l)} \leftarrow \left(\mathcal{E}_1(\mathbf{h}^{(l)}; \mathbf{A}_1), \ldots, \mathcal{E}_c(\mathbf{h}^{(l)}; \mathbf{A}_c)\right)$ ▷ Inner product features (optional)
$\mathbf{z_i} \leftarrow \text{MLP}(\mathbf{h}^{(l)}; \omega)$ ▷ Pass through MLP

1.2 Supplementary Note 2: Orientation ambiguity of local coordinate frames

Note that the tangent space $\mathbb{T}_i$ is defined only up to an orthogonal transformation (rotation and reflection) within the tangent space of $\mathcal{M}$ because the m-dimensional $\mathcal{T}_i\mathcal{M}$ only constrains m coordinates of the frame. However, when the signal is projected to the local frame, the tangent frame alignment by $\mathcal{P}_{j\to i} = \mathbf{O}_{ij}$ removes this ambiguity. Indeed, suppose that each node carries the same signal $\mathbf{f}$, then, parallel transport alignment of the projected signal from j to i yields

$$\mathbb{T}_i^T\mathbf{f} = \mathbf{O}_{ij}\mathbb{T}_j^T\mathbf{f} = (\mathbb{T}_j\mathbf{O}_{ji})^T\mathbf{f} = \mathbb{T}_i^T\mathbf{f}\,, \tag{1}$$

where the first equality used the definition of parallel transport, the second equality used the transpose operation, and the third equality used Eq. 2 in the main article. Note that the same result does not hold when parallel transporting signals in the ambient space (without projection) because, in that case, the ambiguity in the frame orientation introduces ambiguity in the signal.

1.3 Supplementary Note 3: Mathematical construction of the gradient filters

We describe the LFFs using gradient filters, which approximate the variation of the vector field around points. We first numerically compute the gradient of a scalar field and define directional derivates in all orthogonal directions of a local coordinate frame. Then, we extend this concept to the covariant derivative of a vector field by realising that, after parallel transport into a common tangent space, the covariant derivative is just a concatenation of channel-wise gradients.

Formally, we consider the local frame $\mathbb{T}_i$ and construct the directional derivative filter[2] in the direction of the q-th unit vector $\mathbf{t}_i^{(q)}$ (i.e., a weighted message passing operation, Supplementary Fig. 1e). We follow Ref.[2] and decompose $\mathbf{t}_i^{(q)} \in \mathbb{R}^{d\times 1}$ by projecting it to the set of edge vectors $\mathbf{e}_{ij}$ to obtain a vector $\widehat{\mathbf{t}}_i^{(q)} \in \mathbb{R}^{n\times 1}$ at node i

$$\widehat{t}_i^{(q)}(j) = \begin{cases} \langle \mathbf{t}_i^{(q)}, \mathbf{e}_{ij}\rangle / deg(i) & \text{if } j \in \mathcal{N}(i,1) \\ 0 & \text{otherwise}\,. \end{cases} \tag{2}$$

Collating for all nodes, the q-th coordinate of $\mathbb{T}_i$ projected onto the edge vectors is the matrix $\widehat{\mathbb{T}}_q = (\widehat{\mathbf{t}}_1^{(q)}, \dots, \widehat{\mathbf{t}}_n^{(q)}) \in \mathbb{R}^{n \times n}$. The directional derivative of the scalar field s_i at i in the direction $\mathbf{t}_i^q$ then becomes a weighted finite difference over the graph, namely

$$\mathcal{K}^{(i,q)} s_i := \langle \nabla s_i, \widehat{\mathbf{t}}_i^{(q)} \rangle = \sum_{j \in \mathcal{N}(i,1)} (s_j - s_i)\, \widehat{t}_i^{(q)}(j)\,. \tag{3}$$

In matrix form,

$$\mathbf{K}^{(q)} \mathbf{s} = (\widehat{\mathbb{T}}_q - \mathrm{diag}(\widehat{\mathbb{T}}_q \, \mathbf{1}_n))\mathbf{s}\,, \tag{4}$$

where $\mathbf{1}_n$ is the $n \times 1$ vector of ones. As a result, the gradient of a scalar field can be obtained by column-wise concatenating (as new channels) the derivatives against all directions in the basis set

$$\nabla \mathbf{s} = (\mathcal{D}^{(1)} \mathbf{s}, \dots, \mathcal{D}^{(d)} \mathbf{s})\,. \tag{5}$$

Supplementary Fig. 2 shows the output of the first and second-order filters applied to a linear and a quadratic scalar field.

To generalise the gradient to a vector field$\mathbf{F} \in \mathbb{R}^{n \times m}$, one first parallel transports the local frames at the neighbours j to i (Supplementary Fig. 1d) before applying the directional derivative filters (Eq. 4) channel-wise in (Supplementary Fig. 1e). Let $\mathbf{O}$ denote the $nm \times nm$ block matrix of $m \times m$ blocks given by the connection matrices $\mathbf{O}_{ij}$. Then, we may express Eq. 7 in the main article in matrix form as

$$\mathcal{D}^{(q)} \mathbf{F} = ((\mathbf{K}^{(q)} \otimes \mathbf{1}_m^T \mathbf{1}_m) \odot \mathbf{O})\mathbf{F}\,. \tag{6}$$

Here the Kronecker product in the inner brackets expands $\mathbf{K}^{(q)}$ to the $nm \times nm$ block matrix where the (i, j) $m \times m$ block is filled with entries $K_{ij}^{(q)}$.

2 Supplementary Figures

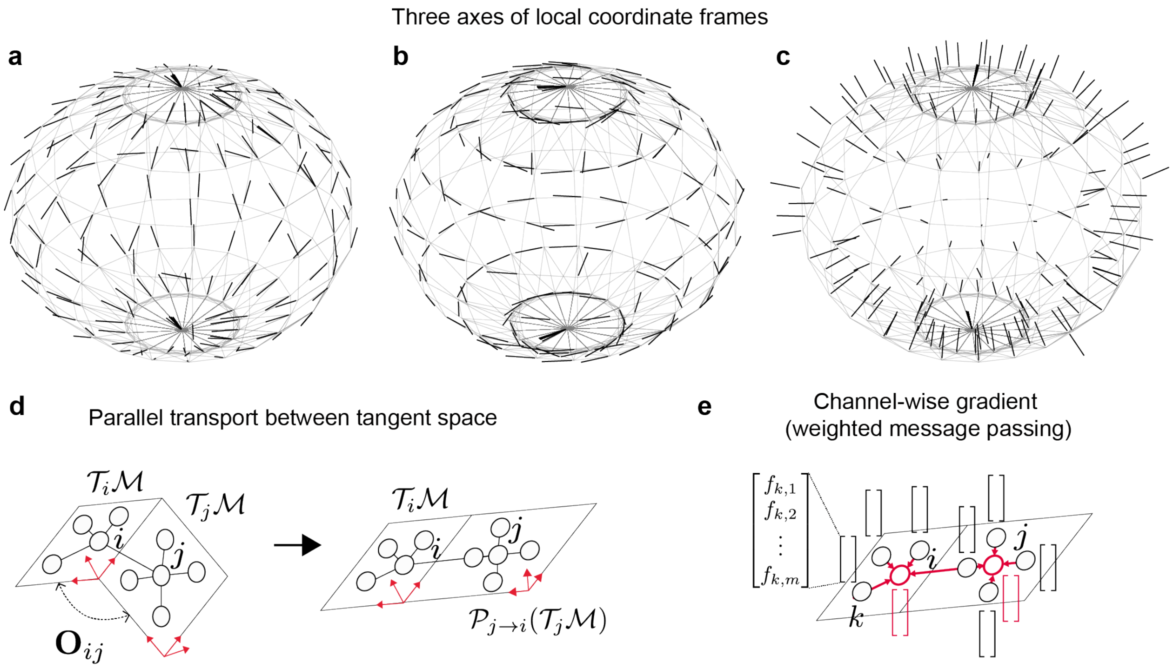

Supplementary Figure 1: **Illustration of mathematical constructions for manifold computations.** Local coordinate frames were fitted to eight neighbours at each point on the grid over a sphere (manifold of dimension two) embedded into $\mathbb{R}^3$. **a,b** Unit vectors representing within-manifold components of the coordinate frame. **c** Unit vectors representing the normal component. Note that the orientation of the normals is not necessarily consistent. **d** Parallel transport maps two adjacent tangent spaces into a common (Euclidean) vector space. **e** In this Euclidean vector space, the covariant derivative operator is equivalent to taking gradients channel-wise. These channel-wise gradients can be approximated by finite differences and computed through message passing.

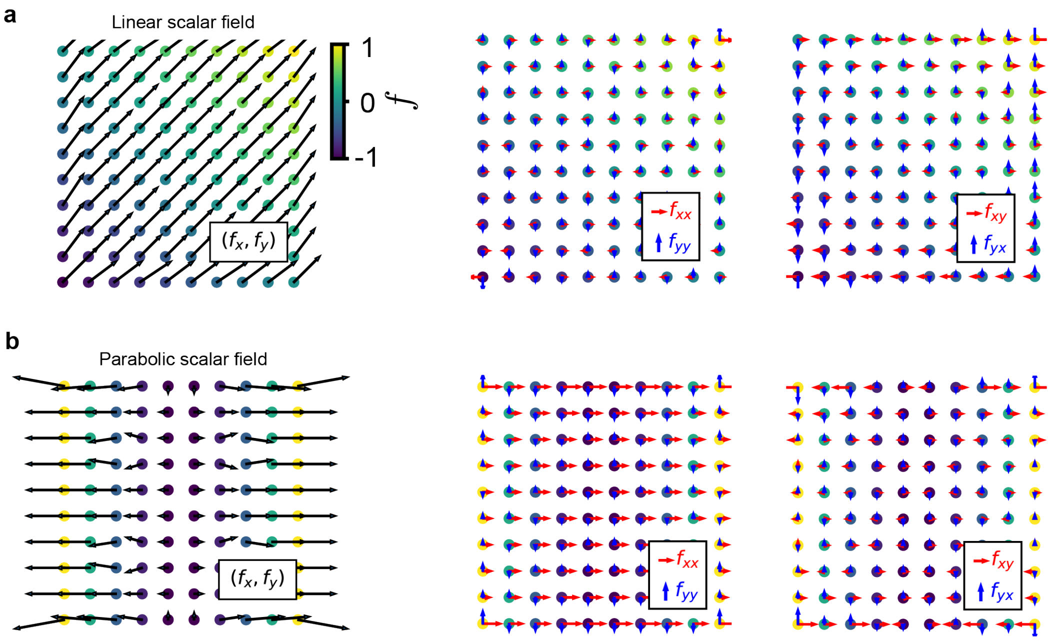

Supplementary Figure 2: **Output of gradient filters.** **a** Example linear scalar field. **b** Example parabolic scalar field. The first column shows the output of the scalar field convolved with gradient filters to approximate directional derivatives in principal spatial coordinates. The second and third columns show second-order mixed partial derivatives obtained by a subsequent second application of the gradient filter to the derivative signal. In each case, we used a uniform rectangular grid with eight neighbours.

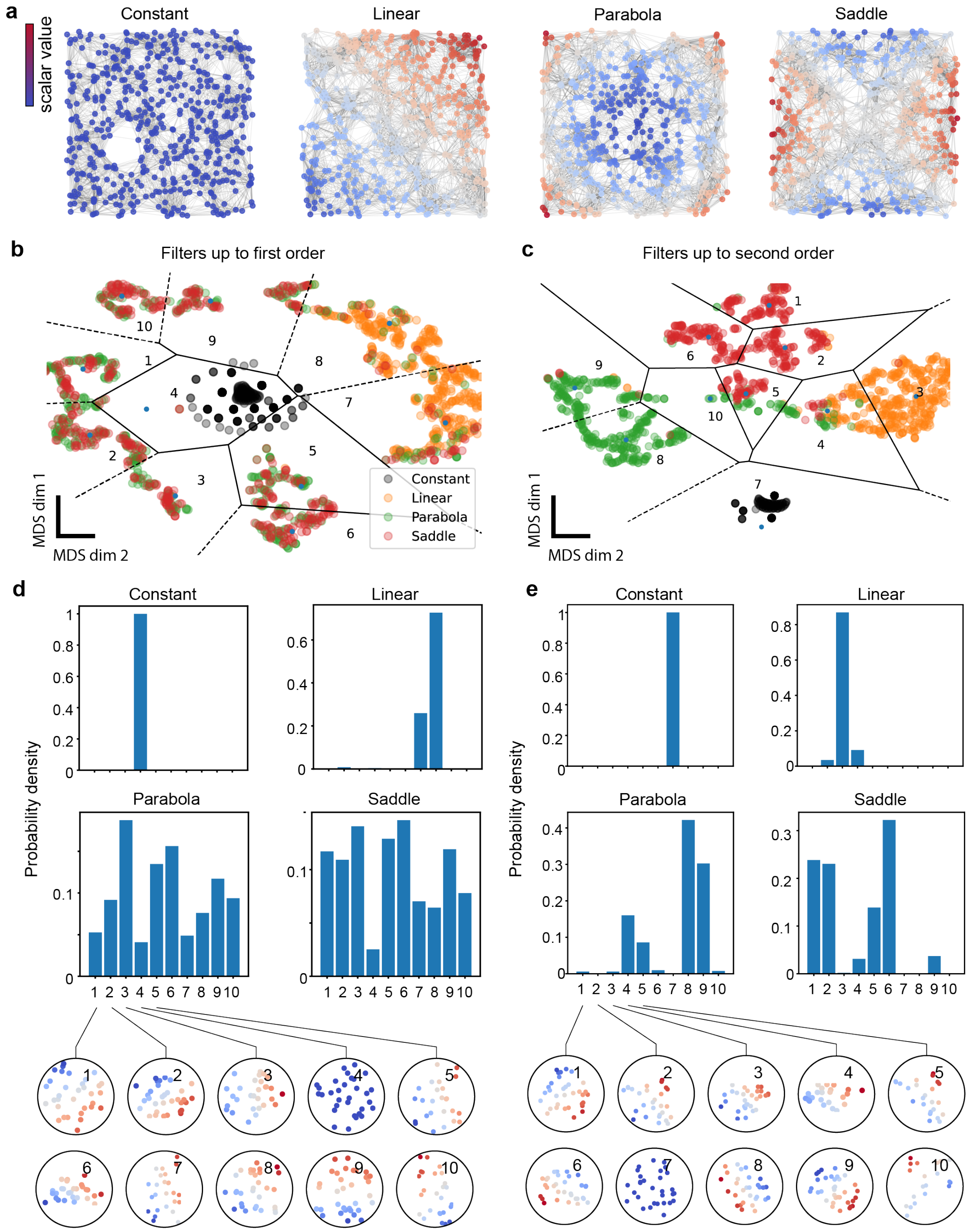

Supplementary Figure 3: **Effect of filter order.** **a** Scalar fields sampled uniformly ($n = 512$) at random in the interval $[-1, 1]^2$ and fitted with a continuous k-nearest neighbour graph (black lines, $k = 20$). **b** Joint MARBLE representation of all LFFs (scalar) based on first-order (1-hop) gradient filters. Dots represent points drawn from a. Close points signify similar LFF (scalar). Black lines show k-means clustering (15 clusters). **c** As in b, but with second-order (2-hop) gradient filters. The increased filter order increases the clustering of features, at the expense of more model parameters. **d** Histogram of neighbourhood types shown as circular insets. **e** As in d, but with second-order gradient filters. The increased filter order better discriminates the parabola and saddle but shows little difference for constant and linear fields.

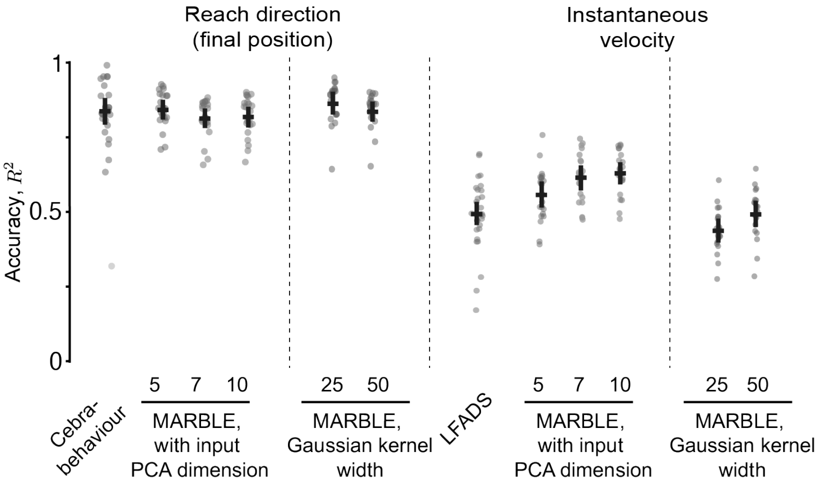

Supplementary Figure 4: **Sensitivity analysis of MARBLE representations for the macaque arm reaching neural data against preprocessing hyperparameters.** Each point represents the average accuracy for a given session for sessions 1-20. For the Gaussian kernel sweep, we used a dataset with five principal components. For comparison, we display the strongest benchmark from Figure 4 on decoding of research direction (Cebra-behaviour) and velocity (LFADS). Horizontal and vertical bars show mean and one std, respectively ($n = 43$).

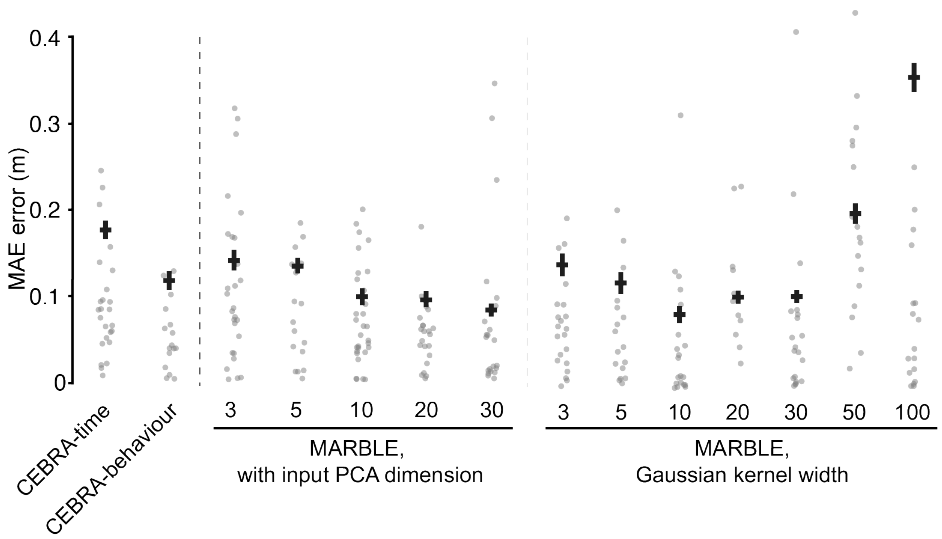

Supplementary Figure 5: **Sensitivity analysis of MARBLE representations for the rat hippocampus neural data against preprocessing hyperparameters.** For benchmarking, we compare the obtained mean absolute errors (MAE) against CEBRA-time (self-supervised) and CEBRA-behaviour (supervised with animal position and running direction as labels). Each point represents a time point. For the Gaussian kernel sweep, we used a dataset with ten principal components. Horizontal and vertical bars show mean and one std, respectively ($n = 2000$).

3 Supplementary Tables

Supplementary Table 1: **Description of hyperparameters used for MARBLE representations.**

Hyperparameter	Use	Role
k	Preprocessing	Number of neighbours in the proximity graph.
delta (δ)	Preprocessing	Density of neighbours in the proximity graph.
spacing (α)	Preprocessing	Spacing of samples relative to manifold. diameters
number_of_resamples	Preprocessing	Number of times the proximity graph is fitted.
frac_geodesic_nb (K)	Preprocessing	Fraction of neighbours used to fit local frames.
order (p)	Feature extraction	Highest derivative order of features.
inner_product_features	Feature extraction	Embedding-aware or -agnostic modes.
diffusion	Feature extraction	Fixed-point preserving denoising of the vector field.
epochs	Training	Number of gradient descent steps.
batch_size	Training	Batch size of the optimisation.
lr	Training	Initial learning rate, later automatically optimised.
momentum	Training	Momentum of the gradient descent.
hidden_channels	Training	Number of hidden channels in the MLP.
out_channels (E)	Latent reps.	Dimension of latent representations.
emb_norm	Latent reps.	Leads to spherical layout to compare with CEBRA.

Supplementary Table 2: **Default hyperparameters and usage.**

**Hyperparameter**	**Default**	**Requires setting**	**Effect**
k	20	–	Increase to obtain a connected graph.
delta (δ)	1.0	Sometimes	Increase to make the LFFs larger and obtain more discriminative representations. In this sense, δ has a similar effect to the minimum distance parameter in UMAP.
spacing (α)	0.015	Sometimes	Subsampling ensures that LFFs are not overrepresented. Increasing leads to less data but more even samples. Set to 0 to facilitate point-by-point decoding.
number_of_resamples	1	–	Increase if the data is sparse.
frac_geodesic_nb (K)	1.5	–	Higher than 1.0 means that also second-degree neighbours are taken.
order (p)	2	–	Increase to obtain more refined features but an exponential increase of parameters. Lower if the network does not converge because of little data.
inner_product_features	False	+	Setting to True the latent representations embedding-agnostic.
diffusion	True	+	Disable if data has little noise to yield increased resolution.
epochs	100	–	Increase if the network has not yet converged (training and validation losses are still decreasing).
batch_size	64	–	Lower to get better generalisation but at the cost of slower convergence.
lr	0.01	–	Lower to get better generalisation but at the cost of slower convergence.
momentum	0.9	–	Increase to get better generalisation at the expense of convergence.
hidden_channels	[32]	Sometimes	Increase for more expressive representations. For best generalisation, find the minimal value where representations do not change. Add multiple layers, e.g., [32, 32] to increase feature capacity.
out_channels (E)	3	+	Increase until the variation of latent variables are captured. Keep low to minimise the number of model weights.
emb_norm	False	Sometimes	Enable to limit the embedding space to the surface of a sphere. We recommend using it only when comparing with CEBRA.

Supplementary Table 3: **Hyperparameters used in the different experiments.** We used embedding aware mode whenever possible for the highest expressivity, except when the manifold embedding varied. The graph density parameter δ was kept at 1.0, except for the real-world datasets, where higher δ resulted in more expressive representations. Subsampling of $\alpha = 0.015$ was used except in the macaque and rat data, we did not subsample ($\alpha = 0$) to facilitate point-by-point decoding. The learnable diffusion was used in the macaque example to denoise the vector field but was disabled in other examples for faster training. Second-order features were used in all examples except the rat dataset, whose small size necessitated using first-order features to reduce the number of parameters. We used 32 hidden channels except for the macaque dataset, where increasing this hyperparameter led to more resolved representations. The latent space dimension E was tuned to maximise the expressivity of the model. In the macaque and rat examples, it was set to match the latent dimension of the benchmark models.

	**Dataset**			
**Parameters**	**van der Pol**	**RNN**	**Macaque**	**Rat**
Embedding aware/agnostic	aware/agnostic	aware/agnostic	aware	aware
Subsampling (α)	0.015	0.015	0.0	0.0
Graph density δ	1.0	1.0	1.4	1.4
Diffusion	False	False	True	False
order (p)	2	2	2	1
hidden_channels	32	32	100	32
out_channels (E)	5	3	3/20	3/32
