# Supplementary material for: MARBLE: interpretable representations of neural population dynamics using geometric deep learning
Source: Nat Methods. 2025 Feb 17;22(3):612–20. doi: 10.1038/s41592-024-02582-2 (PMC11903309; doi:10.1038/s41592-024-02582-2)
Supplement: Supplementary file 2 — Reporting Summary [file 41592_2024_2582_MOESM2_ESM.pdf]

nature portfolio

Corresponding author(s): Adam Gosztolai

Last updated by author(s): Sep 26, 2024

Reporting Summary

Nature Portfolio wishes to improve the reproducibility of the work that we publish. This form provides structure for consistency and transparency in reporting. For further information on Nature Portfolio policies, see our Editorial Policies and the Editorial Policy Checklist.

Statistics

For all statistical analyses, confirm that the following items are present in the figure legend, table legend, main text, or Methods section.

n/a	Confirmed	
☐	☒	The exact sample size (*n*) for each experimental group/condition, given as a discrete number and unit of measurement
☐	☒	A statement on whether measurements were taken from distinct samples or whether the same sample was measured repeatedly
☐	☒	The statistical test(s) used AND whether they are one- or two-sided<br>*Only common tests should be described solely by name; describe more complex techniques in the Methods section.*
☐	☒	A description of all covariates tested
☐	☒	A description of any assumptions or corrections, such as tests of normality and adjustment for multiple comparisons
☐	☒	A full description of the statistical parameters including central tendency (e.g. means) or other basic estimates (e.g. regression coefficient) AND variation (e.g. standard deviation) or associated estimates of uncertainty (e.g. confidence intervals)
☐	☒	For null hypothesis testing, the test statistic (e.g. *F*, *t*, *r*) with confidence intervals, effect sizes, degrees of freedom and *P* value noted<br>*Give P values as exact values whenever suitable.*
☒	☐	For Bayesian analysis, information on the choice of priors and Markov chain Monte Carlo settings
☒	☐	For hierarchical and complex designs, identification of the appropriate level for tests and full reporting of outcomes
☒	☐	Estimates of effect sizes (e.g. Cohen's *d*, Pearson's *r*), indicating how they were calculated

Our web collection on statistics for biologists contains articles on many of the points above.

Software and code

Policy information about availability of computer code

Data collection	The data in this study partly constitutes experimental data from the literature and partly generated by custom code. The code used to generate the synthetic datasets can be found at https://github.com/agosztolai/MARBLE.
Data analysis	The code to carry out the simulations and analysis can be found at https://github.com/agosztolai/MARBLE.

For manuscripts utilizing custom algorithms or software that are central to the research but not yet described in published literature, software must be made available to editors and reviewers. We strongly encourage code deposition in a community repository (e.g. GitHub). See the Nature Portfolio guidelines for submitting code & software for further information.

Data

Policy information about availability of data

All manuscripts must include a data availability statement. This statement should provide the following information, where applicable:

- Accession codes, unique identifiers, or web links for publicly available datasets
- A description of any restrictions on data availability
- For clinical datasets or third party data, please ensure that the statement adheres to our policy

The data generated during the simulations is available here https://doi.org/10.7910/DVN/KTE4PC.

Human research participants

Policy information about studies involving human research participants and Sex and Gender in Research.

Reporting on sex and gender	Not applicable.
Population characteristics	Not applicable.
Recruitment	Not applicable.
Ethics oversight	Not applicable.

Note that full information on the approval of the study protocol must also be provided in the manuscript.

Field-specific reporting

Please select the one below that is the best fit for your research. If you are not sure, read the appropriate sections before making your selection.

☒ Life sciences ☐ Behavioural & social sciences ☐ Ecological, evolutionary & environmental sciences

For a reference copy of the document with all sections, see nature.com/documents/nr-reporting-summary-flat.pdf

Life sciences study design

All studies must disclose on these points even when the disclosure is negative.

Sample size	No explicit a priori sample size calculation was performed since we used publicly available datasets and synthetic data.<br><br>For the experiments involving synthetic data, the sample size was chosen to ensure that the dynamic changes of interest could be reliably detected and statistically evaluated. Specifically, we generated a sufficiently large number of samples (200 trajectories for RNN) to observe the variability of the system across different conditions (changes in gain) and to ensure that the trends were stable and not due to random fluctuations. Since we did not perform a statistical test here - no explicit sample size calculation was necessary.<br><br>For the macaque experimental dataset, we used all available data from the repository https://github.com/nplcode/lfads-neural-stitching-reproduce. The size of this dataset is inherently determined by the number of trials and recordings in the original experimental design. Given that this data has been widely used in the literature and is considered robust, we believe the sample size is sufficient to provide valid insights for our analyses.<br><br>Similarly, for the rat experimental dataset, we used the full dataset available from the repository https://crcns.org/data-sets/hc/hc-11/about-hc-11 As with the macaque data, this dataset represents a comprehensive set of trials designed to capture the behavioral and neural dynamics under study. Thus, the sample size reflects the original experiment's design, which is presumed to be sufficient for detecting the relevant neural or behavioral effects.
Data exclusions	No data was excluded.
Replication	MARBLE neural networks were trained five times. The results obtained on each attempt were consistent and reproducible.
Randomization	Randomization was not applicable to this study because the data used were derived from pre-existing datasets (synthetic data, macaque experimental data, and rat experimental data) collected under controlled experimental conditions. In these datasets, the experimental conditions and variables were already predetermined by the original study design. As such, our study did not involve any intervention or group assignment where randomization would be necessary. Instead, our focus was on analyzing the available data to observe specific neural and behavioral dynamics, which does not require further randomization of experimental groups.
Blinding	Blinding was not applicable to this study because the analysis was conducted on pre-existing datasets (synthetic data, macaque experimental data, and rat experimental data) that had already been collected under controlled conditions. The primary aim of the study was to analyze objective, quantitative data, such as neural and behavioral metrics, which are not subject to subjective interpretation or observer bias. Since the data analysis did not involve subjective assessments, treatment interventions, or human participants, there was no need to implement blinding procedures.

Reporting for specific materials, systems and methods

We require information from authors about some types of materials, experimental systems and methods used in many studies. Here, indicate whether each material, system or method listed is relevant to your study. If you are not sure if a list item applies to your research, read the appropriate section before selecting a response.

Materials & experimental systems

n/a	Involved in the study
☒	☐ Antibodies
☒	☐ Eukaryotic cell lines
☒	☐ Palaeontology and archaeology
☒	☐ Animals and other organisms
☒	☐ Clinical data
☒	☐ Dual use research of concern

Methods

n/a	Involved in the study
☒	☐ ChIP-seq
☒	☐ Flow cytometry
☒	☐ MRI-based neuroimaging
